# Supplementary material for: Analysis of Nuclear Dynamics in Nematode-Trapping Fungi Based on Fluorescent Protein Labeling
Source: J Fungi (Basel). 2023 Dec 11;9(12):1183. doi: 10.3390/jof9121183 (PMC10744682; doi:10.3390/jof9121183)
Supplement: Supplementary file 1 [file jof-09-01183-s001.zip › (annotation) Supplementary Table, Figures and Videos.pdf]

1. Supplementary tables

Table S1. Primers used in this study.

**primers**	**Paired sequences**	**Purpose**
H_2BGFP_pjet/LB_F1	GATCTTCCGGATGGCTCGAGGATGGATTGGAGTAGGTTTGGTG	Verification of construction vector
H_2BGFP_GFP/LB_R1	GCTCCTCGCCCTTGCTCACCATGGCGCGCCTTTTGGCAGACGAAGATGAATACT	
H_2BGFP_LB/GFP_F2	ATTCATCTTCGTCTGCCAAAAGGCGCGCCATGGTGAGCAAGGGCGAGGAG	Verification of construction vector
H_2BGFP_hph/GFP_R2	GACCTCCACTAGCATTACACTTCTACTTGTACAGCTCGTCCATG	
H_2BGFP_GFP/hph_F3	GGACGAGCTGTACAAGTAGAAGTGTAATGCTAGTGGAGGTCAACA	Verification of construction vector
H_2BGFP_RB/hph_R3	GAAACCAAAAAAACTTAATACGCTGGGGGGAGTTTAGGGAAAGAGC	
H_2BGFP_hph/RB_F4	CCTAAACTCCCCCCAGCGTATTAAGTTTTTTTGGTTTCGC	Verification of construction vector
H_2BGFP_pjet/RB_R4	ATTGTAGGAGATCTTCTAGAGTGCAACTTAAACCCTCCGAAG	
H_2B-YZ-F	CCGAACAGAATTTAATCTGA	Verification of construction vector
H_2B-YZ-R	GCGGAGCGAGGATAAGCATTA	
EGFP-Probe-F	ACGTAAACGGCCACAAGTTC	Southern blotting verification
EGFP-Probe-R	TGCTCAGGTAGTGGTTGTCG	
RT-GFP-F	GGTGAACTTCAAGATCCGCC	Analysis of RT-PCR
RT-GFP-R	CTTGTACAGCTCGTCCATGC	
RT-β-tublin-F	TCGGTGGAGGTACTGGTT	Analysis of RT-PCR
RT-β-tublin-R	GAGTGTTCGAGGGTGGTG	

2. Supplementary figures

Figure S1. Analysis of *A. oligospora* digestive nematodes. (**A**) Fluorescence observation of mature nematode captured by fungi in different planes. Yellow arrow: *C. elegans*. Blue arrow: traps. Bar: 100 μm. (**B**) Fluorescence observation of young adult nematode captured by fungi in different planes. Yellow arrow: *C. elegans*. Blue arrow: traps. Bar: 100 μm. (**C**) The traps and captured nematodes of the H_2B positive strains were observed via SEM. Left: Traps captured a large number of nematodes. Yellow arrow: the captured nematodes. Blue arrow: conidium. Bar: 500 μm. Right: An enlarged schematic of the red dashed box in the left image. Through SEM analysis, it was found that the mycelium had entered the nematode body. Red arrow: the outline of nematode. Bar: 50 μm. (**D**) Hyphae were stained with CFW, and traps of the H_2B positive strains were observed. Red arrow: septum.

Figure S2. Comparison of mycelial growth between wild type (WT) and H_2B positive strains. (**A**) Colonial morphologies of WT and H_2B positive strains incubated on PDA, TYGA, TG, CMY and CMA medium at 28°C for 5 days. Due to the sparse hyphae on CMA media, the edges of the colonies are marked with red circles. (**B**) The mycelial growth rate of WT and H_2B positive strains cultured on five different media in Figure S2A. The number after H_2B represents three independent H_2B positive strains.

Figure S3. Comparison of conidiation, trap formation and nematicidal activity in WT and H_2B positive strains. (**A**) The conidial yields of WT and H_2B positive strains. (**B**) Comparison of the spore germination of WT and H_2B positive strains at 4, 8, and 12 h. (**C**) Comparison of traps produced by WT and H_2B positive strains. (**D**) Comparison of nematode mortality caused by WT and H_2B positive strains. Error bars represent the standard deviations. Ns: No significant difference (Tukey's HSD, $P > 0.05$).

Figure S4. Observation of the formation process of traps of H_2B positive transformants. (**A**) Observation the induction of H_2B positive transformants by nematodes to produce a large number of traps. White arrow: traps. Bar: 50μm. (**B**) Based on Figure S4A, SEM observations were conducted on the hyphae, traps, and captured nematodes. WT data unshown. Red arrow: conidium. Yellow arrow: septum. (**C**) Observation of the formation process of traps. White arrow: traps. Red arrow: the extended trap cell loop. Bar: 100 μm. (**D**) Fluorescence observation after 48 hours of spore germination. White arrow: traps. Blue arrow: hypha. Bar: 200 μm.

Figure S5. Analysis of conidiophore and germination tube of H_2B positive strains. (**A**) Morphological observation of conidium and conidiophore via white light. Bar: 200 μm. (**B**) The conidium and conidiophore were observed laterally by fluorescence microscope. Bar: 100 μm. (**C**) Observation of conidium and conidiophore from above. Bar: 100 μm. Yellow arrow: conidiophore. Red arrow: conidium. (**D**) Conidium sprouted a mycelium. (**E**) Conidium sprouted two hyphae. (**F**) Conidium sprouted three hyphae. Bar: 50 μm.

Red arrow: conidium. Black arrow: hyphae.

Figure S6. Analysis of nuclear localization at the tip of hyphae. (**A**) Fluorescence observation of the nucleus at the tip of the hyphae. Yellow arrow: septum. Red arrow: the nucleus near the tip of the hyphae. Bar: 25 μm. (**B**) The nucleus migrates towards the tip of the hyphae. (**C**) The nucleus migrates in the opposite direction to the tip of the hyphae. (**D**) After nuclear migration, there is a nucleus at the tip of the hyphae. (**E**) Analysis of nuclear localization at the tip of branching hyphae. Blue arrow: direction of nuclear migration. Red arrow: the nucleus near the tip of the hyphae. White triangle: the tip of the hyphae. Bar: 100 μm.

3. Supplementary videos

Video S1. Observation of traps capturing nematodes. Red arrow: traps. Yellow arrow: nematode eggs. Bar: 500 μm. The video was played at 100× speed.

Video S2. Spatial imaging analysis of traps based on EGFP.

Video S3. Analysis of nuclear behavior interaction between conidium and hyphal cells. Red arrow: conidium. Bar: 200 μm. The video was played at 100× speed.

Video S4. The nuclei evenly distributed in the mycelium were moving. Bar: 100 μm. White dotted line: hyphal outline. The video was played at 100× speed.

Video S5. Observation of nuclear division in hypha. Red arrow: the nucleus involved in division. Bar: 100 μm. The video was played at 150× speed.

Video S6. Nuclear behavior in the hyphal cells of H_2B-EGFP-labeled strain. The nucleus could cross the adjacent nucleus and migrated to the tip of hypha. Red arrow: the nucleus that was crossed. Bar: 100 μm. The video was played at 100× speed.

Video S7. Analysis of nuclear migration in branching hyphae. Red arrow: migrated nucleus. Bar: 60 μm. White dotted line: hyphal outline. The video was played at 100× speed.

Video S8. Rapid migration of nuclei in mycelium. Red arrow: Rapidly migrated nuclei. Bar: 50 μm. White dotted line: hyphal outline. The video was played at 100× speed.
