# Supplementary figures and images for: Analysis of Nuclear Dynamics in Nematode-Trapping Fungi Based on Fluorescent Protein Labeling
Source: J Fungi (Basel). 2023 Dec 11;9(12):1183. doi: 10.3390/jof9121183 (PMC10744682; doi:10.3390/jof9121183)

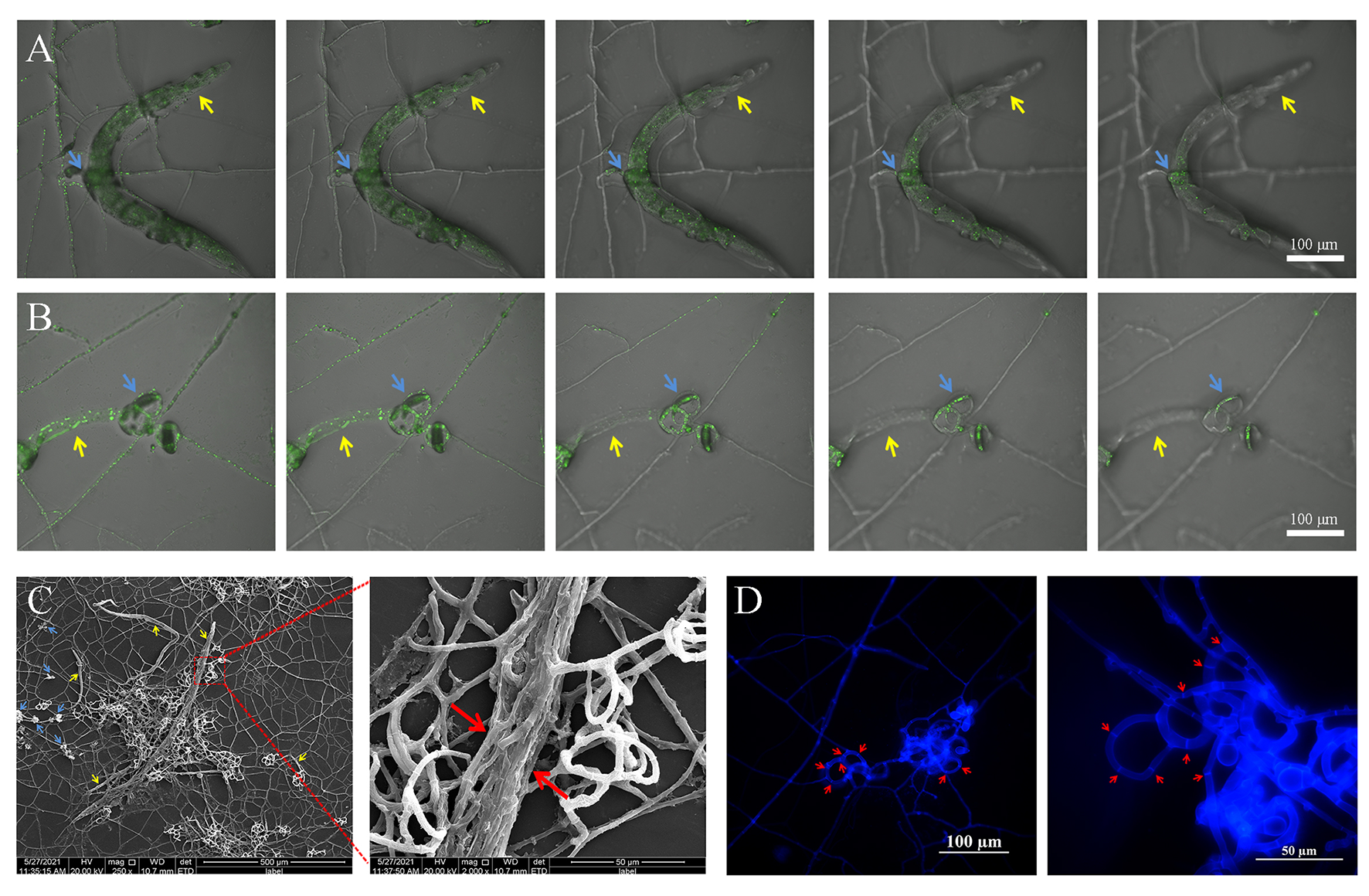

Supplement: Supplementary file 1 [file jof-09-01183-s001.zip › Supplementary Figure S1.tif]

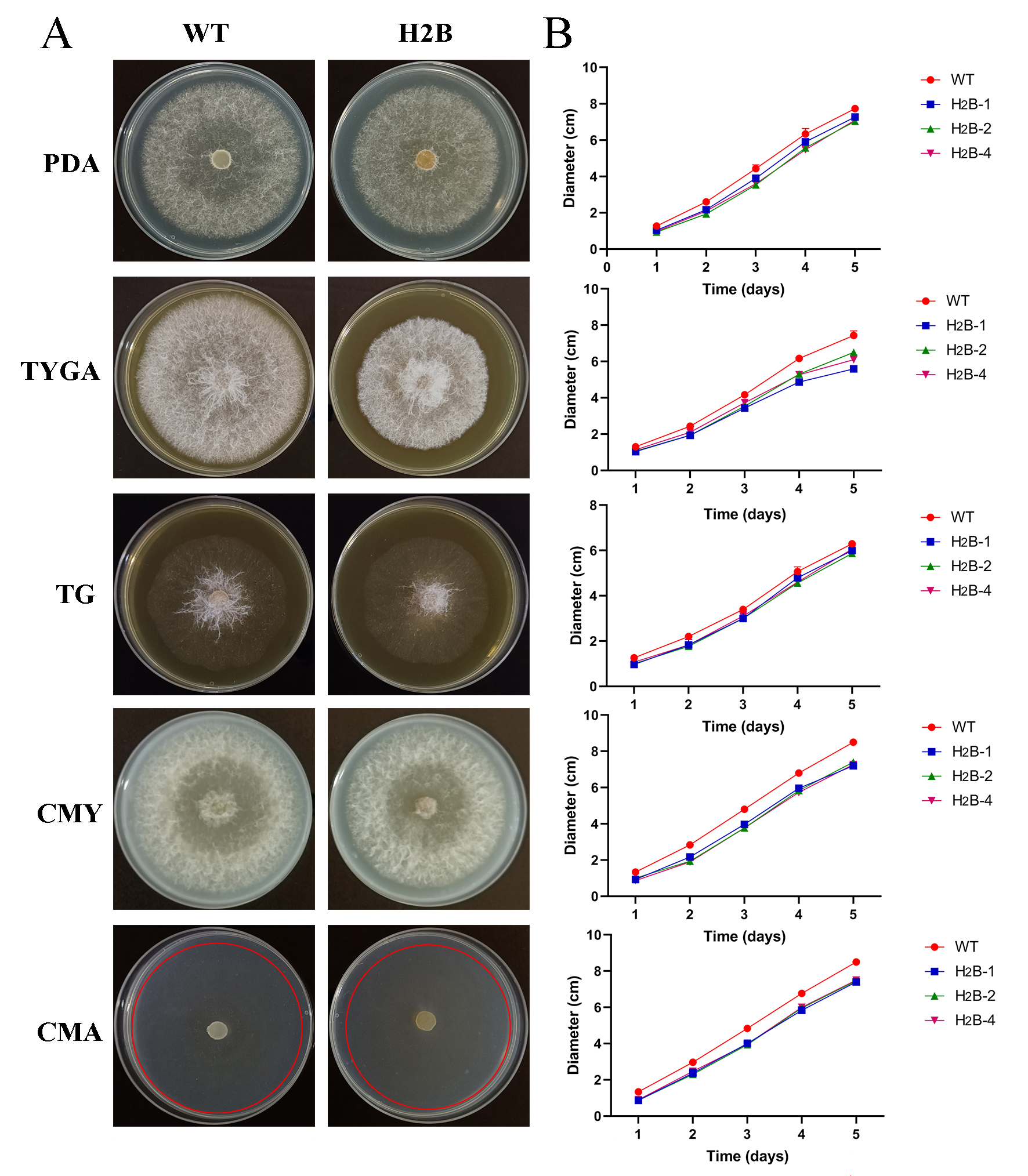

Supplement: Supplementary file 1 [file jof-09-01183-s001.zip › Supplementary Figure S2.tif]

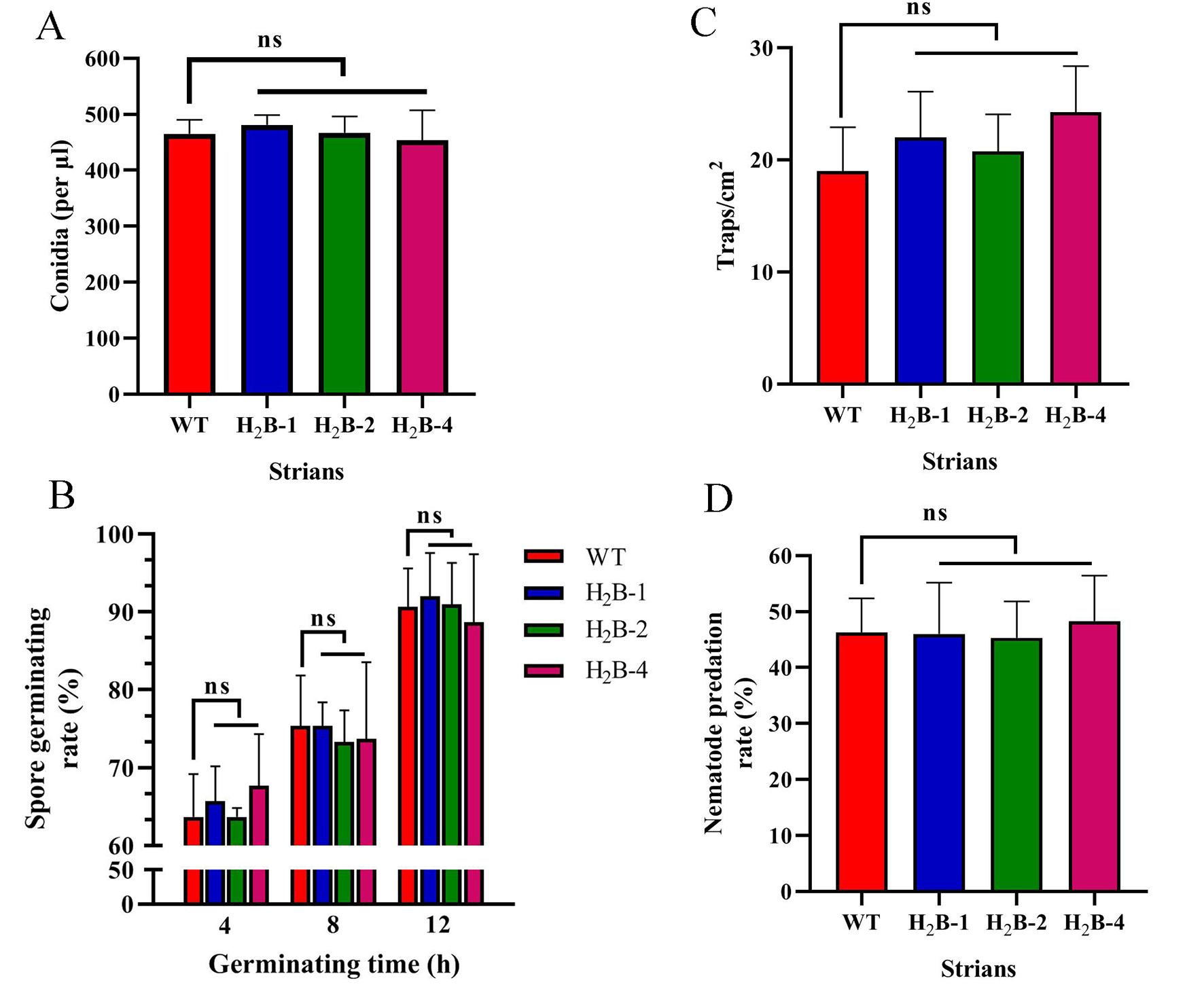

Supplement: Supplementary file 1 [file jof-09-01183-s001.zip › Supplementary Figure S3.tif]

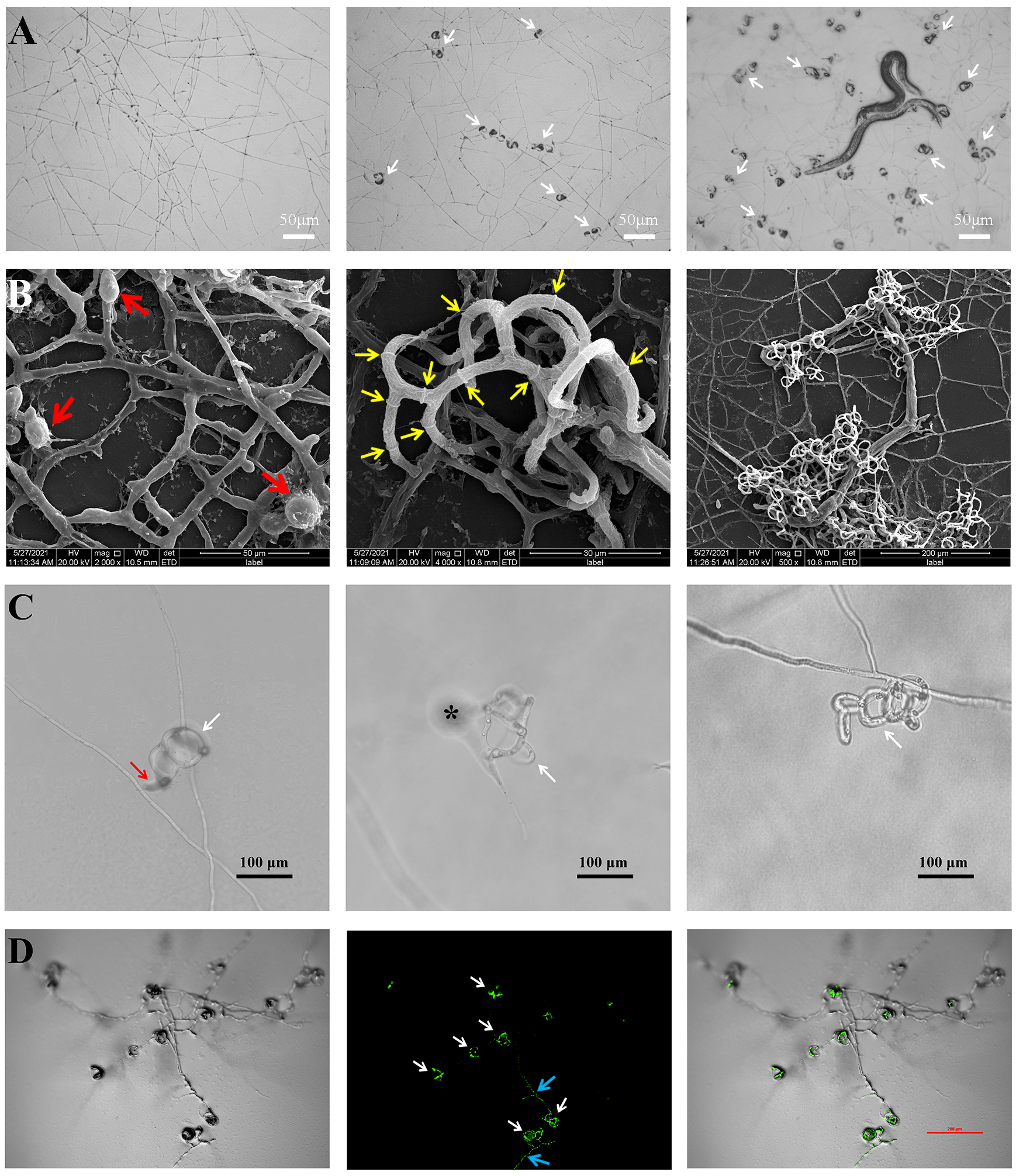

Supplement: Supplementary file 1 [file jof-09-01183-s001.zip › Supplementary Figure S4.tif]

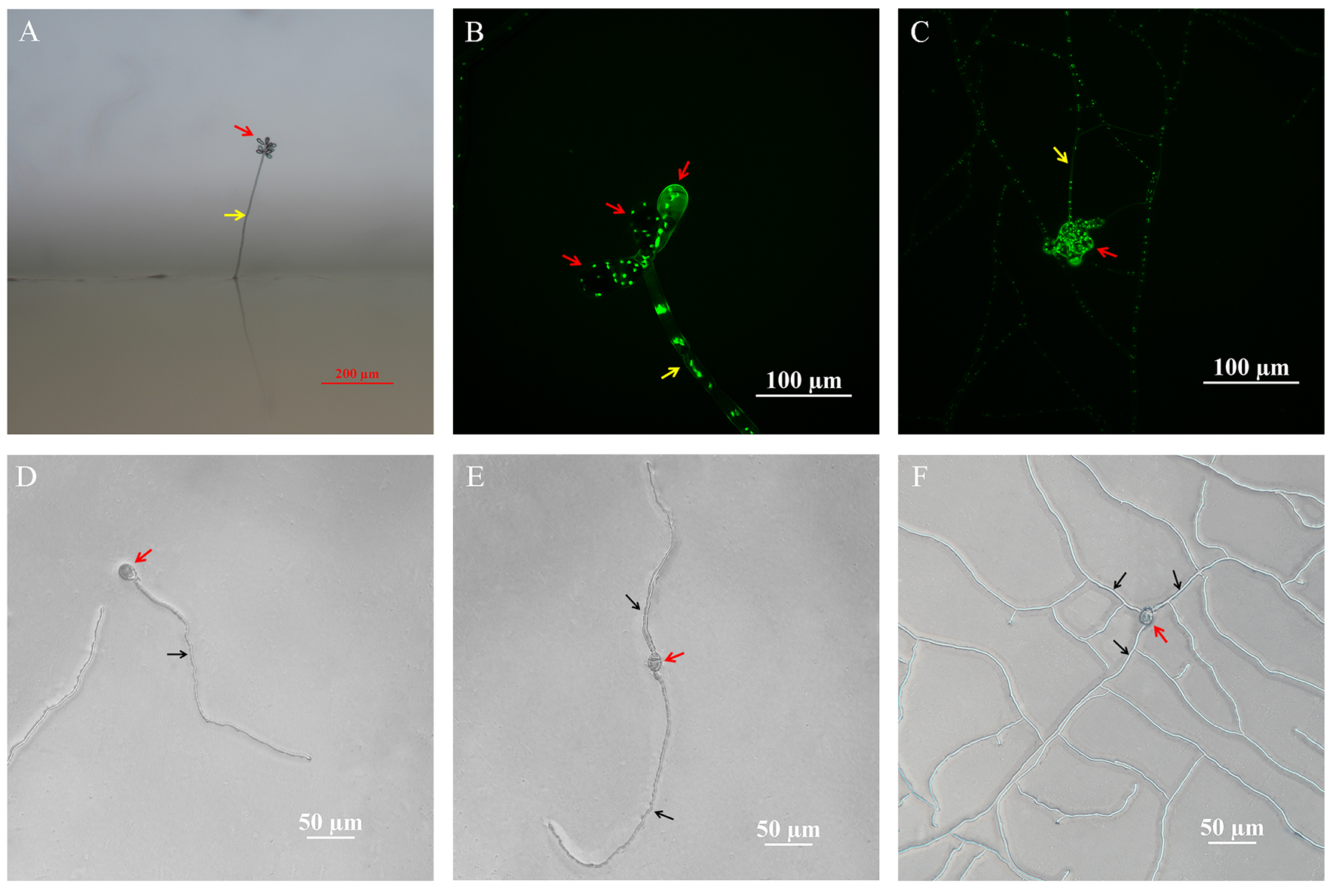

Supplement: Supplementary file 1 [file jof-09-01183-s001.zip › Supplementary Figure S5.tif]

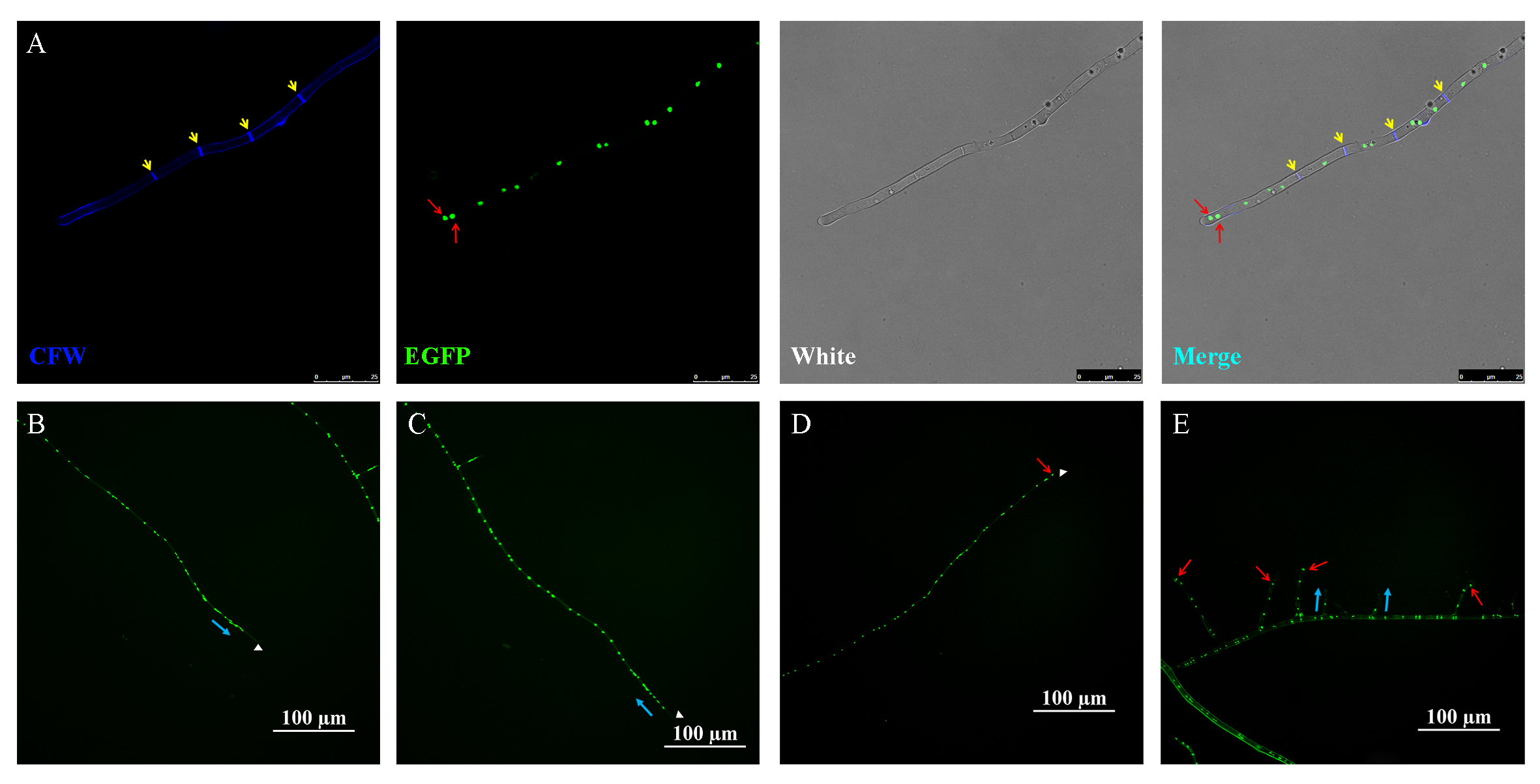

Supplement: Supplementary file 1 [file jof-09-01183-s001.zip › Supplementary Figure S6.tif]
